# Targeted Therapy With PI3K and FGFR Inhibitors on Human Papillomavirus Positive and Negative Tonsillar and Base of Tongue Cancer Lines With and Without Corresponding Mutations

**DOI:** 10.3389/fonc.2021.640490

**Published:** 2021-05-11

**Authors:** Stefan Holzhauser, Nicole Wild, Mark Zupancic, Ramona G. Ursu, Cinzia Bersani, Anders Näsman, Ourania N. Kostopoulou, Tina Dalianis

**Affiliations:** ^1^ Department of Oncology-Pathology, Karolinska Institutet, Stockholm, Sweden; ^2^ Department of Microbiology, University of Medicine and Pharmacy, Grigore T. Popa Iasi, Iaşi, Romania

**Keywords:** head neck cancer, HPV, tonsillar cancer, base of tongue cancer, oropharyngeal cancer, FGFR, PI3K, targeted therapy

## Abstract

**Objectives:**

Human papillomavirus positive (HPV^+^) tonsillar and base of tongue squamous cell carcinoma (TSCC/BOTSCC), the major subsites of oropharyngeal squamous cell carcinoma (OPSCC) have favorable outcome, but upon relapse, outcome is poor and new therapies needed. Since, phosphatidyl-inositol-4,5-bisphosphate 3-kinase, catalytic subunit alpha (PIK3CA) and fibroblast-growth-factor-receptor-3 (FGFR3) mutations often occur in such tumors, here, we tested targeted therapy directed to such genes in TSCC/BOTSCC cell lines. We also combined the two types of inhibitors with each other, and cisplatin or docetaxel that are used clinically.

**Methods:**

The HPV^+^ CU-OP-2, -3, -20, UPCI-SCC-154, and HPV^-^ CU-OP-17 and UT-SCC-60A cell lines were first tested for common PIK3CA/FGFR3 mutations by competitive-allele-specific TaqMan-PCR. They were then treated with the food and drug administration (FDA) approved drugs, alpelisib (BYL719) and erdafitinib (JNJ-42756493) alone and in combination with cisplatin or docetaxel. Viability, proliferation, apoptosis and cytotoxicity responses were thereafter followed by WST-1 assays and the IncuCyte S3 Live^®^ Cell Analysis System.

**Results:**

HPV^+^ CU-OP-2 had a pS249C-FGFR3, and like CU-OP-20, a pE545K-PIK3CA mutation, while no other lines had such mutations. Irrespectively, dose dependent responses to all PI3K/FGFR inhibitors were obtained, and upon combining the inhibitors, positive effects were observed. Cisplatin and docetaxel also induced dose dependent responses, and upon combination with the inhibitors, both positive and neutral effects were found.

**Conclusions:**

The data suggest that FDA approved drugs alpelisib and erdafitinib efficiently inhibit TSCC/BOTSCC cell line growth, especially when combined irrespective of presence of corresponding mutations and should be further explored, for use upon recurrent disease.

## Introduction

Human papillomavirus positive (HPV^+^) tonsillar and base of tongue squamous cell carcinoma (TSCC/BOTSCC), the dominant oropharyngeal squamous cell carcinoma (OPSCC) subsites, generally have better outcome than corresponding HPV-negative (HPV^-^) cancer and their incidences have increased (1–12). To develop personalized medicine for these patients, attempts have been made to find prognostic biomarkers, of which most, but not all are defined by immunohistochemistry (12–26). By next generation sequencing, phosphatidylinositol-4,5-bisphosphate 3-kinase, catalytic subunit alpha (PIK3CA) and fibroblast growth factor receptor 3 (FGFR3) mutations were however frequently revealed in HPV^+^ TSCC/BOTSCC (27–29). FGFR3 overexpression has also been reported in HPV^+^ TSCC/BOTSCC/OPSCC, and in addition, FGFR3 and PI3K3CA mutations have been associated to poorer prognosis (29–32). Therapies with FGFR and phosphoinositide 3-kinases (PI3K) inhibitors have been used for other cancers with FGFR3 and PIK3CA mutations, and so we tested the HPV^+^ UM-SCC-47 and UPCI-SCC-154, and the HPV^-^ UT-SCC-60A cell lines for sensitivity to FGFR inhibitor AZD4547, and PI3K inhibitors BEZ235 and BKM120 (33–35). None of these cell lines displayed the most common FGFR3/PIK3CA mutations, but all exhibited dose dependent sensitivity, and upon combining the inhibitors, synergy was observed (35). Since then, the Food and Drug Administration (FDA) has approved the PI3K and FGFR inhibitors alpelisib (BYL719) and erdafitinib (JNJ-42756493) resp (36, 37). and we now have cell lines with PIK3CA and FGFR3 mutations (see below).

Consequently, here the effects of FDA approved alpelisib and erdafitinib were examined on HPV^+^ UPCI-SCC-154, and HPV^-^ UT-SCC-60A, and the newly established HPV^+^ CU-OP-2, -3, -20 and HPV^-^ CU-OP-17 lines, of which two had FGFR3 and/or PIK3CA mutations. We also combined these inhibitors with each other, previous inhibitors, as well as cisplatin and docetaxel, two chemotherapeutic drugs used clinically for TSCC/BOTSCC therapy.

## Material and Methods

### Cell Lines and Seeding

HPV^+^ UPCI-SCC-154 and HPV^-^ UT-SCC-60A were provided by S.Gollin, University of Pittsburgh USA, and R.Grénman, University of Turku, Finland resp. and their culture conditions described before (38, 39). HPV^+^ CU-OP-2, CU-OP-3, CU-OP-20 and HPV^-^ CU-OP-17, with wild type p53, were provided by N.Powell, Cardiff University UK, and grown on 60 Gy irradiated 3T3 fibroblasts as feeder layers as described before (40, 41). For analysis, UPCI-SCC-154 and UT-SCC-60A, with 5000 cells/well, and CU-OP lines (without feeders), with 7500 cells/well, were seeded in 96-well plates in 90 - 200 μl.

### Competitive Allele-Specific TaqMan PCR (CAST-PCR)

Competitive Allele-Specific TaqMan^®^ PCR technology (Thermo Fischer Scientific, Waltham, MA, USA) was used exposing reference FGFR3 gene and its variants p.R248C, p.S249C and p.K650Q and reference PIK3CA gene and its variants p.E542K, p.E545K and p.H1047R (30, 35, 42).

### Drugs and Treatments

BYL719, BEZ235, AZD4547 and JNJ-42756493 (Selleckhem Chemicals Munich, Germany) stocks were obtained either already diluted in DMSO, or dissolved upon arrival in DMSO, according to the instructions of the manufacturer. Stock solutions were at 10 mM for all inhibitors, except BEZ235 where a stock solution of 2 mM was recommended. Further dilutions were done in PBS, and were for: AZD4547 5-25 μM; JNJ-42756493 0.01-10 μM; BEZ235 0.25-1 μM; and for BYL719 0.5-20 μM. Cisplatin (Accord Healthcare Limited, Middlesex, UK) was diluted in PBS and used at 2.5-7.5 μM. Docetaxel (Actavis, Hafnarfjordur, Island) was diluted in PBS and used at 0.6-6 nM. All experiments were repeated minimum three times.

### WST-1 Viability Assay

Viability was quantified using the WST-1 assay (Roche Diagnostics, Mannheim, Germany) and followed for 72 h according to the instructions of the manufacturer and before (35, 42).

### Cell Proliferation, Apoptosis, and Cytotoxicity Assays

Cells in 96-well plates, were placed into the IncuCyte S3 Live^®^ Cell Analysis System, using the IncuCyte Caspase-3/7 Green Apoptosis Assay Reagent and the Incucyte™ Cytotox Red Reagent (Essen Bioscience, Welwyn Garden City, UK), and followed for proliferation, apoptosis and cytotoxicity by images taken every 2 h (42).

### Statistical Analysis

To verify effects of single or combination treatments compared to the negative control, a multiple t test accompanied by correction for multiple comparison of the means conferring to the Holm Sidak method was done (35, 42, 43). The combined effects were evaluated applying the effect-based approach ‘Highest Single Agent’ (44), where details were presented previously (35, 42, 43).

## Results

### Detection of FGFR3 and PIK3CA Mutations in HPV+ and HPV- Cell Lines

The three most common FGFR3 and PIK3CA mutations were analyzed by CAST-PCR as described before (30, 38). CU-OP-2 had a pS249C (FGFR3) and a pE545K (PIK3CA) mutation, CU-OP-20 presented only the latter, while CU-OP-3 and -17 had no such mutations, similar to that shown before for HPV^+^ UPCI-SCC-154 and HPV^-^ UT-SCC-60A (35).

### Viability After Single Exposure to PI3K and FGFR Inhibitors in HPV+ and HPV- Cell Lines

Dose dependent responses to BYL719, BEZ235, JNJ-42756493 and AZD4547 compared to PBS when tested by WST-1 assays are shown below for the CU-OP lines. For HPV^+^ UPCI-SCC-154 and HPV^-^ UT-SCC-60A only data on BYL719-JNJ-42756493 are presented, since data on BEZ235-AZD4547 were published before (35).

**Figure 1:**
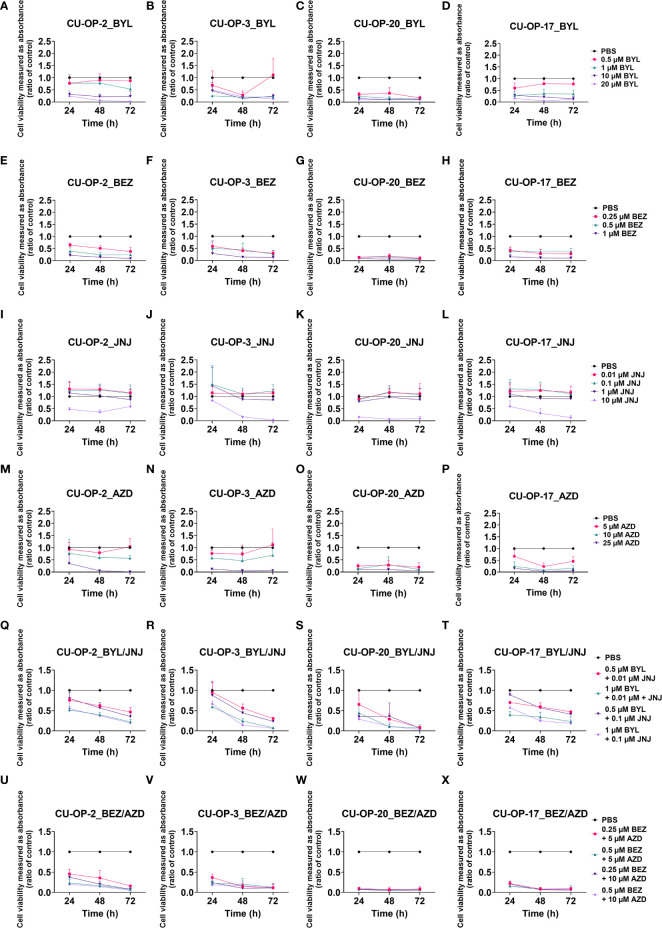
WST-1 viability assays evaluating cellular metabolic capacity by absorbance on HPV^+^ CU-OP-2, -3, -20 and HPV^-^ CU-OP-17 cell lines upon treatment with PI3K inhibitors (BYL719, BEZ235), and FGFR inhibitors (JNJ-42756493, AZD4547). WST-1 viability assays measuring absorbance following treatment for 24, 48 and 72 h of HPV+ CU-OP-2, -3, -20 and HPV- CU-OP-17 with PI3K and FGFR inhibitors, BYL719 **(A–D)**; BEZ235 **(E–H)**; JNJ-42756493 **(I–L)**; and AZD4547 **(M–P)**; and combinational treatments with BYL719 and JNJ-42756493 **(Q–T)**; as well as BEZ235 and AZD4547 **(U–X)**. The graphs represent three experimental runs per cell line, and results are presented as the mean ± standard deviation. BYL denotes BYL719; BEZ denotes BEZ235; AZD denotes AZD4547 and JNJ denotes JNJ-42756493.

*In all CU-OP cell lines, c*ompared to PBS, the highest BYL719 dose (20 μM) decreased viability consistently (p<0.01) (**Figures 1A–D**). Declines in viability were also obtained with 1-10 μM BYL719 for all most time points (except continually for CU-OP-2 with 1 μM, and CU-OP-3 with 10 μM after 24 h), while 0.5 µM BYL719, decreased viability only in CU-OP-20 (for all indicated, at least p<0.01) (**Figures 1A–D**). BEZ235 at all doses decreased viability in all lines the whole 72 h period when compared to PBS, except for CU-OP-2 and CU-OP-3 with 0.5 μ after 24 h (for all others, at least p<0.05) (**Figures 1E–H**). Notably, only the highest dose JNJ-42756493 reduced viability significantly compared to PBS in all CU-OP lines, at most time points (except CU-OP-2 at 72 h and CU-OP-3 at 24 h) (for all, at least p<0.05) (**Figures 1I–L**). Likewise, only the highest AZD4547 dose (25 µM) decreased viability in all CU-OP lines compared to PBS the whole 72 h period, however for HPV^+^ CU-OP-20 and HPV^-^ CU-OP-17, this also the case for 5-10 µM AZD4547 at 48 and 72 h (for all, at least p<0.01), while CU-OP-2 and -3 were more resistant (**Figures 1M–P**).

**Figure 2:**
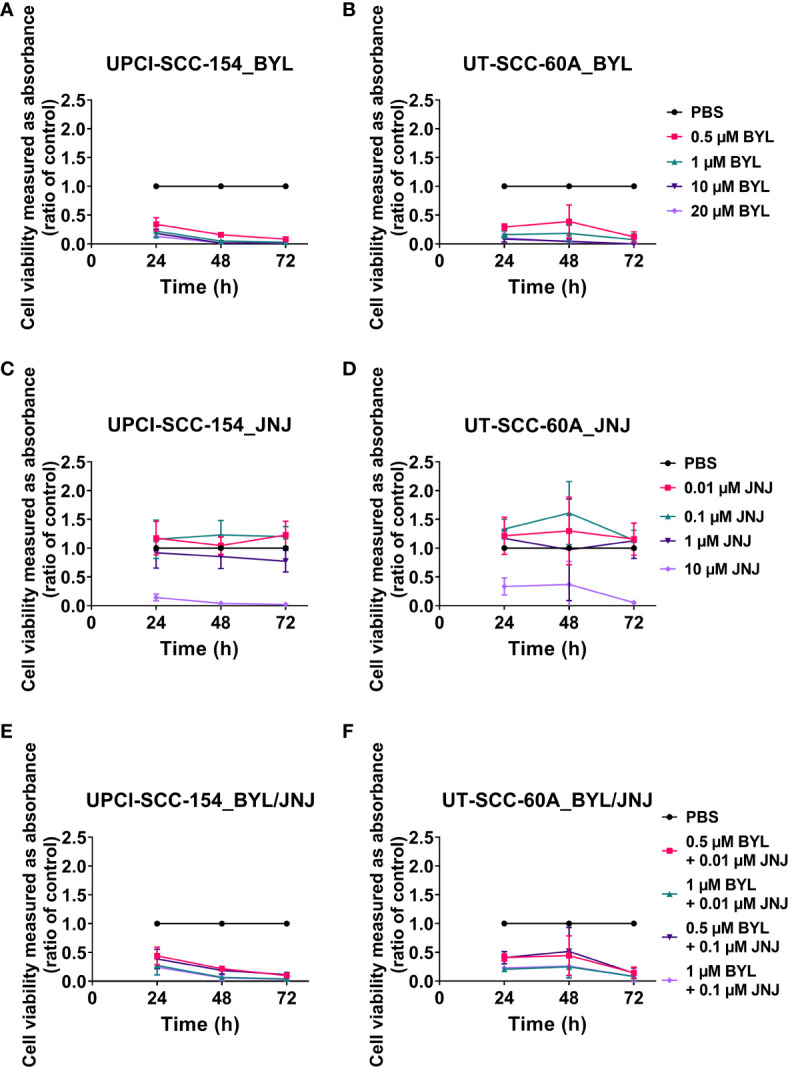
WST-1 viability assays evaluating cellular metabolic capacity by absorbance on HPV^+^ UPCI-SCC-154 and HPV^-^ UT-SCC-60A cell lines upon treatment with PI3K inhibitor BYL719, and FGFR inhibitor JNJ-42756493. WST-1 viability assay measuring absorbance following treatment for 24, 48 and 72 h of HPV^+^ UPCI-SCC-154 and HPV^-^ UT-SCC-60A with PI3K and FGFR inhibitors, BYL719 **(A, B)** and JNJ-42756493 **(C, D)** as well as combinational treatment with BYL719 and JNJ-42756493 **(E, F)**. The graphs represent three experimental runs per cell line, and the data are presented with the mean ± standard deviation. BYL denotes BYL719 and JNJ denotes JNJ-42756493.

*In HPV^+^ UPCI-SCC-154 and HPV^-^ UT-SCC-60*, BYL719 decreased viability compared to PBS at all time points with all doses (at least p<0.01), (**Figures 2A, B**), while this was only the case with the highest JNJ-42756493 dose (10 µM) (for all, at least p<0.05) (**Figures 2C, D**).

To summarize, all cell lines showed dose dependent responses to all inhibitors, with IC_50_ values between 0.04-1.74 μM for BYL719 and 2.34-13.74 μM for JNJ-42756493 at 72 h (**Table 1**). CU-OP-20 and UPCI-SCC-154 were generally more sensitive to all inhibitors, while CU-OP-2 (with both PIK3CA and FGFR3 mutations) was more resistant, and the remaining cell lines could be sensitive to one, and more resistant to another inhibitor (**Table 1**).

**Table 1:** Estimation of inhibitory concentration 50% (IC_50_) based on WST–1 viability analysis following treatment with the FGFR inhibitor (JNJ-42756493 and AZD4547), PI3K inhibitors (BYL719 and BEZ235), and the chemotherapeutic drugs cisplatin and docetaxel for 24, 48 and 72 h.

	*IC_50_ per time (h)^a^*		
	24 h	48 h	72 h
**CU-OP-2**			
BYL^d^	4.09	3.08	1.74
JNJ^d^	11.97^b^	7.6	13.74^b^
BEZ^d^	0.36	0.21	0.15
AZD^d^	22.96	10.2	12.37
Cisplatin^d^	<0.001^c^	8.78^b^	3.75
Docetaxel^e^	2695^b^	39.55^b^	19.5^b^
**CU-OP-3**			
BYL^d^	6.56	0.24	1.48
JNJ^d^	84.89^b^	3.56	2.81
BEZ^d^	0.42	0.23	0.13
AZD^d^	10.45	7.93	16.29
Cisplatin^d^	<0.001^c^	<0.001^c^	7.78
Docetaxel^e^	<0.001^c^	<0.001^c^	<0.001^c^
**CU-OP-20**			
BYL^d^	0.28	0.26	0.13
JNJ^d^	2.66	3.58	3.02
BEZ^d^	0.06	0.07	0.03
AZD^d^	1.69	2.73	1.26
Cisplatin^d^	<0.001^c^	24.62^b^	6.71
Docetaxel^e^	<0.001^c^	2.45	0.72
**CU-OP-17**			
BYL^d^	0.63	0.99	0.93
JNJ^d^	17.25^b^	5.83	3.57
BEZ^d^	0.23	0.15	0.15
AZD^d^	5.85	1.32	3.06
Cisplatin^d^	<0.001^c^	3.71	0.79
Docetaxel^e^	<0.001^c^	2.83	0.39
**UPCI-SCC-154**			
BYL^d^	0.29	0.08	0.04
JNJ^d^	3.78	2.82	2.34
Cisplatin^d^	<0.001^c^	14.15^b^	2.26
Docetaxel^e^	11.27^b^	1.40	0.26
**UT-SCC-60A**			
BYL^d^	0.21	0.29	0.07
JNJ^d^	8.43	7.81	4.32
Cisplatin^d^	<0.001^c^	4.44	3.91
Docetaxel^e^	1.40	0.19	0.16

^a^The inhibitory concentration 50% (IC50) for each cell line for each drug was determined from log concentrations effect curves in GraphPad Prism using nonlinear regression analysis. ^b^Extrapolated IC50 value, i.e., outside the tested concentration range. ^c^The IC50 value could not be determined; lowest/highest tested concentration closest to the IC50 is reported. ^d^Micromolar (µM). ^e^Nanomolar (nM)

### Viability After Combined Exposure of PI3K and FGFR Inhibitors in HPV+ and HPV- Cell Lines

All CU-OP lines were exposed to BYL719-JNJ-42756493, and BEZ235-AZD4547 combinations (excluding the highest resp. dose). For UPCI-SCC-154 and UT-SCC-60A only data on the FDA approved drugs are shown, since data on the others have been published (35).

*In all CU-OP lines*, all BYL719-JNJ-42756493 combinations reduced viability compared to PBS, except at 24 h with the 0.5 µM BYL719 and 0.01 µM JNJ-42756493 combination for CU-OP-3, and CU-OP-20, and the 0.5 and 0.1 µM resp. combination for CU-OP-3 and CU-OP-17 (for all remaining, at least p<0.05) (**Figures 1Q–T**). Of note, was the increased sensitivity of the inhibitor-resistant CU-OP-2 line (**Figure 1Q**). All BEZ235-AZD4547 combinations decreased viability compared to PBS in all CU-OP lines (for all, at least p<0.01) (**Figures 1U–X**).

*In HPV^+^ UPCI-SCC-154 and HPV^-^ UT-SCC-60A*, all BYL719-JNJ-42756493 combinations decreased viability compared to PBS (except 0.5 µM BYL719 and 0.1 µM JNJ-42756493 at 48 h for UT-SCC-60A), for all remaining, at least p<0.05 (**Figures 2E, F**).

**Figure 3:**
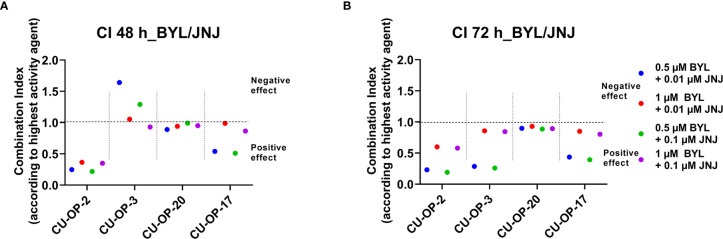
Combinational effects of PI3K inhibitors BYL719 and FGFR inhibitor JNJ-42756493 in HPV^+^ CU-OP-2, -3, -20 and HPV^-^ CU-OP-17 cell lines after 48 and 72 h. Combination indexes (CIs) were shown with the highest single agent approach after 48 h **(A)** and 72 h **(B)**, where CI>1 shows a negative combination effect and CI<1 shows a positive combination effect. CIs were calculated from the mean of three experiments analyzed by WST-1, at 48 and 72 h after treatment. CI denotes combination index; BYL denotes BYL719 and JNJ denotes JNJ-42756493.

To further dissect the effects of inhibitor combinations, combinational indexes (CIs) of BYL719-JNJ-42756493 were calculated for all lines 24-72 h after treatment as done before (35, 42–44). The CIs according to “highest single agent” approach for the lowest combinational doses after 48 and 72 h are shown in **Figure 3** for the CU-OP lines. Positive (CI<1), or neutral (CI=1) combinational effects on the decrease of viability compared to single drugs were observed at 48 h, especially for CU-OP-2 and CU-OP-17, but not for CU-OP-3, where the treatment effect increased after 72 h (**Figure 3**). For UPCI-SCC-154 and UT-SCC-60A mainly neutral effects were obtained (data not shown). For BEZ235 and AZD4547 a similar analysis disclosed positive effects at 24 and 48 h after treatment for CU-OP-2, -3 and -17, the more resistant cell lines (data not shown), and was reported before for UPCI-SCC-154 and UT-SCC-60A (35).

To summarize, combining PI3K-FGFR inhibitors, generally resulted in positive combinatory effect in most cell lines.

### Viability After Single Cisplatin and Docetaxel Exposure Measured in HPV+ and HPV- Cell Lines

**Figure 4:**
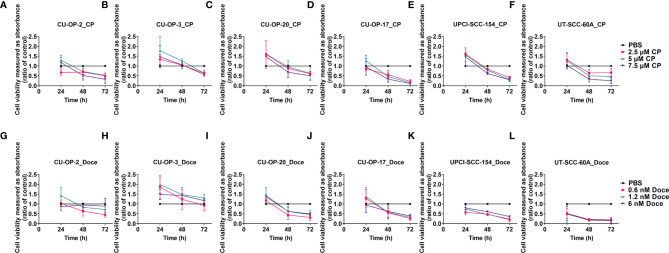
WST-1 viability assays evaluating cellular metabolic capacity by absorbance on HPV^+^ CU-OP-2, -3, -20, and UPCI-SCC-154 and HPV^-^ CU-OP-17 and UT-SCC-60A cell lines upon treatment with cisplatin and docetaxel. WST-1 viability assays measuring absorbance following treatment for 24, 48 and 72 h of HPV^+^ CU-OP-2, -3, -20 and UPCI-SCC-154 and HPV- CU-OP-17 and UT-SCC-60A with cisplatin **(A–F)** and docetaxel **(G–L)** resp. The graphs represent three experimental runs per cell line, and the data are presented with the mean ± standard deviation. CP denotes cisplatin and DOCE denotes docetaxel.

All cells lines presented dose dependent responses to single treatments with cisplatin or docetaxel as shown with WST-1 assays in **Figure 4** and with IC50 values in **Table 1**.

All CU-OP lines presented decreased viability compared to PBS with 2.5-7.5 µM of cisplatin 72 h after treatment, except for CU-OP-20 with 5 µM cisplatin (for all remaining, at least p<0.05) (**Figures 4A–D**). This was also the case, after 48 h, for CU-OP-2 with 7.5 µM cisplatin, and for CU-OP-17 with 5 and 7.5 µM cisplatin (for all, at least p<0.05) (**Figures 4A, D**). All CU-OP lines showed dose dependent responses to docetaxel, but only CU-OP-20 and CU-OP-17 displayed decreased viability compared to PBS with all doses at 72 h (at least p<0.05), as was the case for CU-OP-2 with 6 nM docetaxel (p<0.05) (**Figures 4G–K**).

HPV+ UPCI-SCC-154 and HPV- UT-SCC-60A had dose dependent responses to 2.5-7.5 µM cisplatin compared to PBS, and decreased viability 48 and 72 h after treatment with the 7.5 µM dose, as was the case with the 5 µM dose for UT-SCC-60A (for all, at least p<0.05) (**Figures 4E, F**). Both also showed consistent decreased viability after treatment with 0.6-6 nM docetaxel compared to PBS, except at 24 h with all doses (for all others, at least p<0.05) (**Figures 4K, L**).

To summarize, all cell lines had dose dependent responses to cisplatin and docetaxel, with IC50 values at 72 h between 0.79-7.78 μM for cisplatin and 0.16-19.5 nM for docetaxel (**Table 1**). UT-SCC-60A and UPCI-SCC-154 were generally more sensitive than the CU-OP lines (**Figure 4**, **Table 1**).

### Viability Upon BYL719 and JNJ-42756493 and Cisplatin and Docetaxel Combinations in CU-OP Cell Lines

Data combining FDA approved BYL719 or JNJ-42756493 with cisplatin or docetaxel were pursued only with the CU-OP lines, since UPCI-SCC-154 and UT-SCC-60A were with exception of CU-OP-20, slightly more sensitive to cisplatin and docetaxel.

**Figure 5:**
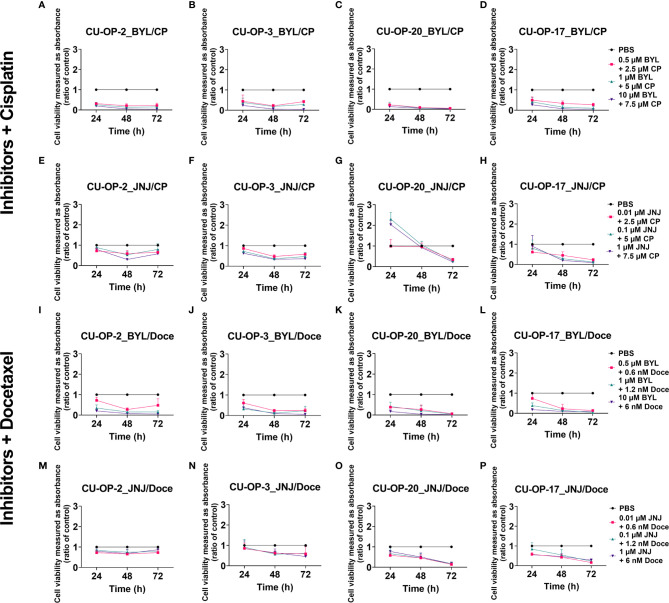
WST-1 viability assays evaluating cellular metabolic capacity by absorbance on HPV^+^ CU-OP-2, -3, -20, and HPV^-^ CU-OP-17 cell lines upon treatment with BYL719 or JNJ-42756493 with cisplatin and docetaxel. WST-1 viability assays measuring absorbance following treatment for 24, 48 and 72 h of HPV^+^ CU-OP-2, -3, -20 and HPV^-^ CU-OP-17 with cisplatin together with BYL719 **(A–D)** or JNJ-42756493 **(E–H)** resp., as well as docetaxel together with BYL719 **(I–L)** or JNJ-42756493 **(M–P)** resp. The graphs represent three experimental runs per cell line, and the data are presented with the mean ± standard deviation. CP denotes cisplatin; DOCE denotes docetaxel; BYL denotes BYL719; JNJ denotes JNJ-42756493.

All BYL719-cisplatin and BYL719-docetaxel combinations decreased viability consistently compared to PBS for all CU-OP lines, except the 0.5 µM BYL719 and 0.6 nM docetaxel combination for CU-OP-2 at 24 and 72 h, and for CU-OP-17 at 24 h (for all remaining, at least p<0.05) (**Figures 5A–D, I–L** resp.). This was also the case for all JNJ-42756493-cisplatin combinations at 72 h after treatment for all CU-OP lines; except for CU-OP-2 with 0.1 µM JNJ-42756493 and 5 µM cisplatin (for all remaining at least p<0.05) (**Figures 5E–H**). CU-OP-17 was the most sensitive line to the JNJ-42756493-cisplatin combination with decreased viability compared to PBS with all doses and time points, except at 24 h for all dose combinations (for all remaining at least p<0.05) (**Figure 5H**). For JNJ-42756493-docetaxel, CU-OP-20 and CU-OP-17 showed decreased sensitivity compared to PBS, 48 and 72 h after treatment, while this was only occasionally the case for CU-OP-2 and CU-OP-3 with the highest dose combinations (for all remaining, at least p<0.05) (**Figures 5M–P**).

Combinational indexes (CIs) of BYL719 and/or JNJ-42756493 with cisplatin or docetaxel were calculated for all cell lines 48-72 h after treatment (35, 38–40), and the CIs after 48 h are presented for the CU-OP cell lines in **Supplementary Figure 1**. Positive (CI<1) or relatively neutral (C~1) effects according to the “highest single agent” approach was dominant for most cell lines, and most prominent for CU-OP-2 and -17 (**Supplementary Figure 1**).

To summarize, mainly positive and neutral effects were obtained upon combining BYL719 and JNJ-42756493 resp. with cisplatin and docetaxel on CU-OP lines, with the best effects observed for CU-OP-2 and CU-OP-17.

### Proliferation, Apoptosis, and Cytotoxicity After Single or Combined Treatment With PI3K, FGFR Inhibitors of HPV+ and HPV- CU-OP Cell Lines

**Figure 6:**
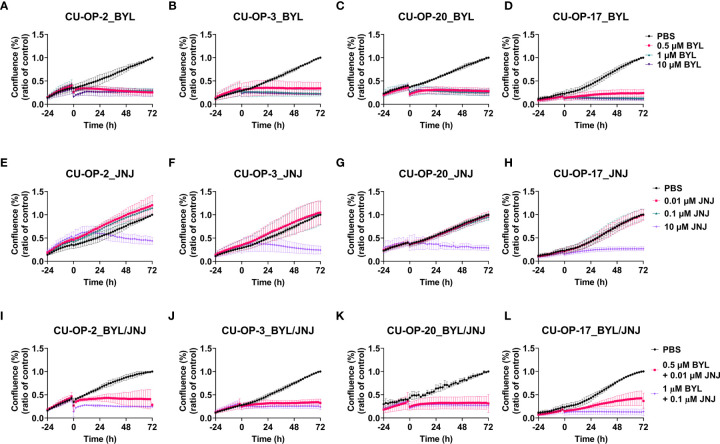
Proliferation response of HPV^+^ CU-OP-2, -3, -20 and HPV^-^ CU-OP-17 cell lines upon treatment with BYL719 and JNJ-42756493. Proliferation responses of HPV^+^ CU-OP-2, -3, -20 and HPV- CU-OP-17 after treatment with PI3K and FGFR inhibitors, BYL719 **(A–D)**; and JNJ-42756493 **(E–H)**; and combinational treatment with BYL719 and JNJ-42756493 **(I–L)**. The graphs represent three experimental runs per cell line. Confluence (%) denotes proliferation response; BYL denotes BYL719; JNJ denotes JNJ-42756493; CP denotes cisplatin; and DOCE denotes docetaxel.

Effects on proliferation, apoptosis and cytotoxicity by single and combined BYL719 and JNJ-42756493 treatments on the CU-OP lines were followed by the IncuCyte S3 Live−Cell Analysis System. All lines showed complete inhibition of proliferation with all BYL719 doses, and the highest JNJ-42756493 dose, and upon combined exposures excluding the highest doses of both inhibitors, proliferation was inhibited with all combination doses (**Figure 6**).

Cytotoxicity was observed for all CU-OP lines with the two highest BYL719 (1 and 10 µM) and the highest JNJ-42756493 (10 µM) concentrations. Upon combined exposure, cytotoxicity was observed in most lines, especially those including 1 µM BYL719 (**Supplementary Figure 2**). Marked apoptosis in all CU-OP lines was present only with the highest JNJ-42756493 dose (10 µM) and not prominent in combinations, where the highest JNJ-42756493 dose was excluded (**Supplementary Figure 3**).

To summarize, all CU-OP lines showed decreased proliferation after single and combined treatments with PI3K and FGFR inhibitors. Cytotoxicity was observed with the two highest BYL719 and the highest JNJ-42756493 doses, and apoptosis was only observed with the latter.

### Proliferation, Apoptosis, and Cytotoxicity After Treatment With Single Chemotherapeutic Agents, or Chemotherapeutic Agents Combined With PI3K and FGFR Inhibitors, on CU-OP Cell Lines

The effects of cisplatin and docetaxel alone, or combined with BYL719 and JNJ-42756493 on proliferation, apoptosis and cytotoxicity in the CU-OP lines were followed. At the doses used both cisplatin and docetaxel inhibited proliferation completely, while neither induced prominent cytotoxicity or apoptosis (data not shown).

Combining BYL719 (0.5-10 µM) or JNJ-42756493 (0.01-1 µM) with either 7.5 µM cisplatin or 6 nM docetaxel, inhibited proliferation completely (data not shown). Cytotoxicity was observed with the two highest BYL719 (1 and 10 µM) and the highest JNJ-42756493 (10 µM) doses with cisplatin or docetaxel for CU-OP-2 and -3, while CU-OP-20 and CU-OP-17 were generally less sensitive especially when docetaxel was present with JNJ-42756493 (data not shown). Prominent apoptosis was only observed in combinations including the highest JNJ-42756493 dose (10 µM) (data not shown).

## Discussion

In this study, the FDA approved drugs alpelisib and erdafitinib induced dose dependent effects with decreased viability and proliferation on HPV+ and HPV- TSCC/BOTSCC cell lines, irrespective of whether the cells had FGFR3 and/or PIK3CA mutations. Furthermore, upon combining these inhibitors mainly positive or neutral effects were disclosed with comparable decreases in viability and proliferation using lower doses of the inhibitors. All tested TSCC/BOTSCC cell lines above also presented dose-dependent effects with diminished viability and proliferation when treated with cisplatin and docetaxel often used clinically, although e.g., CU-OP-2 and CU-OP-3 were relatively more resistant. Subsequently, only the CU-OP lines, were followed for their responses to targeted inhibitor-chemotherapeutic drug combinations, and here also positive and neutral effects were disclosed.

The data on the FDA approved drugs alpelisib and erdafitinib paralleled the inhibitory effects on viability and proliferation, as well as the effects on cytotoxicity and apoptosis of recently tested inhibitors BEZ235 and AZD4547 on UPCI-SCC-154 and UT-SCC-60A (35), suggesting on-target effects of alpelisib and erdafitinib. Both may therefore be of interest for future clinical evaluation in patients with recurrent TSCC/BOTSCC, or for patients with TSCC/BOTSCC, with an estimated risk for a very poor prognosis, due to their tumors disclosing several poor prognostic markers (12, 45). The data also suggest that patients, with tumors both with/without PIK3CA or FGFR3 mutations could respond to the above inhibitors, and that it is not possible upfront to expect that patients with tumors exhibiting PIK3CA or FGFR3 mutations, will respond better or worse to the resp. corresponding inhibitor.

That the cell lines above had drug dependent dose decreases in viability and proliferation, to FDA approved inhibitors, and that effects were enhanced upon combining the two was anticipated, since UPCI-SCC-154 and UT-SCC-60A had responded in an analogous way, to corresponding inhibitors BEZ235 and AZD4547 (35). Notably, HPV+ CU-OP-20 and UPCI-SCC-154 tended to be the most sensitive cell lines to both the older and newer PI3K and FGFR inhibitors, while CU-OP-2 and CU-OP-3 often were less affected. However, upon combination treatments with BYL719 and JNJ-42756493, all TSCC/BOTSCC lines were sensitive.

The enhanced efficacy on inhibition of viability and proliferation, upon combining BYL719 and JNJ-42756493 was in line with previous reports in other cell types, suggesting that PI3K and FGFR inhibitors indeed can be used together and have synergistic effects (35, 42, 43, 46). Furthermore, apart from the positive effects on decreases in viability and proliferation upon PI3K-FGFR inhibitor combinatory treatments, lower doses of the drugs could be used, thereby potentially decreasing side effects and risk of resistance development.

That also BYL719, at the doses used, and not only JNJ-42756493 showed effects on cytotoxicity, was not in line with our previous report, where the AZD4547 (FGFR inhibitor) was superior to the included PI3K inhibitors with regard to inducing cytotoxicity (35, 42, 43). However, the effects on apoptosis were analogous to our previous reports, with JNJ-42756493, an FGFR inhibitor being superior to BYL719, a PI3K inhibitor (35). Nonetheless, joining all data, they definitely support that BYL719 and JNJ-42756493 could be of potential clinical interest for treatment of recurrent TSCC/BOTSCC.

Furthermore, all TSCC/BOTSCC cell lines, with/without FGFR or PI3K mutations, were sensitive to PI3K and FGFR inhibitors and those with such mutations were not necessarily more sensitive or resistant to the inhibitors, similar to that reported for other tumor types (35, 42, 43, 46–53). More specifically, other tumors/tumor lines have also responded to PI3K and FGFR inhibitors, without having PI3K and/or FGFR mutations or chromosomal rearrangements, and having corresponding mutations did not consistently induce increased sensitivity (35, 37, 42, 43, 46–53). So far, we have no explanation to why the TSCC/BOTSCC lines tested here differed in their sensitivity to the included PI3K and FGFR inhibitors. However, it is of note that the two PI3K and FGFR inhibitor most resistant lines CU-OP-2 and CU-OP-3 were previously demonstrated to be radioresistant, while the more sensitive CU-OP-20, was radiosensitive (40).

The HPV+ and HPV- TSCC/BOTSCC cell lines were also examined for their responses to single cisplatin and docetaxel therapy and also here CU-OP-2 and -3 were the most resistant ones, while the remaining cell lines tended to be more sensitive. This pattern was not reflected upon by the effects of cisplatin and docetaxel on proliferation, which was largely inhibited in a similar way for all cell lines. At the doses used however, neither cisplatin or docetaxel disclosed prominent effects on cytotoxicity or apoptosis, but better effects may have been obtained if higher concentrations or repeated treatments had been used. Nonetheless, here we aimed to use as low doses as possible, in order to disclose combinational effects upon combining the chemotherapeutic agents with the inhibitors.

When examining potential combinational effects using cisplatin and docetaxel together with BYL719 or JNJ-42756493, mainly neutral and positive, with occasional negative effects were disclosed. Of note were some positive combinations with BYL719 and cisplatin or docetaxel especially on CU-OP-2, but also with the other cell lines, and the data suggest it could be worthwhile elaborating further on optimizing the effects of such combinations.

To our knowledge, the pursuit of potential positive effects of alpesilib and erdafitinib upon combination with cisplatin and docetaxel have not been examined before in TSCC/BOTSCC, and there are only limited studies on other cell lines, comprising some of the drug-inhibitor combinations in this report. One study on nasopharyngeal cancer cell lines, showed neutral or limited positive effects upon combining BYL719 and cisplatin, while another report on a non-small lung cancer cell, showed more positive effects (40, 54). However, when using corresponding combinations in other cell lines, we obtained not only positive and neutral, but also negative effects [personal communication]. The latter can possibly partially explained by that BYL719 may induce G0/G1 arrest, and thereby may have inhibited some of the cytostatic effects of e.g., cisplatin (55, 56).

This report has limitations. Despite six cell lines were included, more could have been examined. Nonetheless, UPCI-SCC-154 and UT-SCC-60A have extensively been used and the CU-OP cell lines, have the benefit of including lines with PIK3CA and FGFR3 mutations (38–41). Irrespectively, the data do suggest that drug-drug interactions of PI3K and FGFR inhibitors with chemotherapeutic agents could likely be used for treatment of TSCC/BOTSCC. However, future studies, using wider dose ranges, establishing optimal incubation times, and in which sequence the drugs should be administered, as well as mechanistic studies would be useful to accumulate information regarding how to design the best anti-tumor efficacy for clinical use. Still, the chemotherapeutic drug and inhibitor doses tested here are in line with those used by others (36, 52, 54–57).

To conclude, this study supports the potential of further exploring the combined use of FDA approved drugs alpelisib and erdafitinib for the treatment of recurrent TSCC/BOTSCC especially when other options e.g., check point inhibitors are not useful.

## Data Availability Statement

The original contributions presented in the study are included in the article/**Supplementary Material**. Further inquiries can be directed to the corresponding authors.

## Author Contributions

SH and OK did the majority of the experiments, interpreted the data, calculated the statistics and contributed to the writing of the manuscript. CB performed the CAST-PCR to detect the mutations of the cell lines presented in this study. NW and RU collaborated with SH and OK and performed some experiments contributed together with SH and OK in the graphs of the manuscript. NW and RU initiated the experiments and the interpretation of the initial experiments and contributed to the writing of the material and methods section, all under the supervision of SH. MZ and AN assisted in the analysis of the data and in their clinical interpretation. TD, SH, and OK made substantial contributions to conception and design, acquisition of data, analysis and interpretation of data and have been involved in drafting the manuscript and revising it critically for important intellectual content. TD has also provided the sources of the performance of the experiments. TD, OK, SH, MZ, and RU provided the financial support for conducting the research project. All authors contributed to the article and approved the submitted version.

## Funding

This research was funded by the Swedish Cancer Foundation (grant no. 18 0440), the Stockholm Cancer Society (grant no. 181053), the Swedish Cancer and Allergy Foundation (grant no. 190), the Royal Swedish Academy of Sciences (grant no. 2017-2018), the Stockholm City Council (grant no. 20180037), Karolinska Institutet (grant no. 2018:0007) and Stiftelsen Clas Groschinsky Minnesfond (grant no. M19376, and M2025), Lindhes Advokatbyrå (LA2019-0143; LA2020-0077; LA2020-0070), and University of Medicine and Pharmacy Grigore T Popa Iasi (grant no. 6983/21.04.2020).

## Conflict of Interest

The authors declare that the research was conducted in the absence of any commercial or financial relationships that could be construed as a potential conflict of interest.
