# Supplementary material for: Targeted Therapy With PI3K and FGFR Inhibitors on Human Papillomavirus Positive and Negative Tonsillar and Base of Tongue Cancer Lines With and Without Corresponding Mutations
Source: Front Oncol. 2021 May 11;11:640490. doi: 10.3389/fonc.2021.640490 (PMC8144504; doi:10.3389/fonc.2021.640490)
Supplement: Supplementary file 1 [file DataSheet_1.docx]

Supplementary Material

## Supplementary Figures


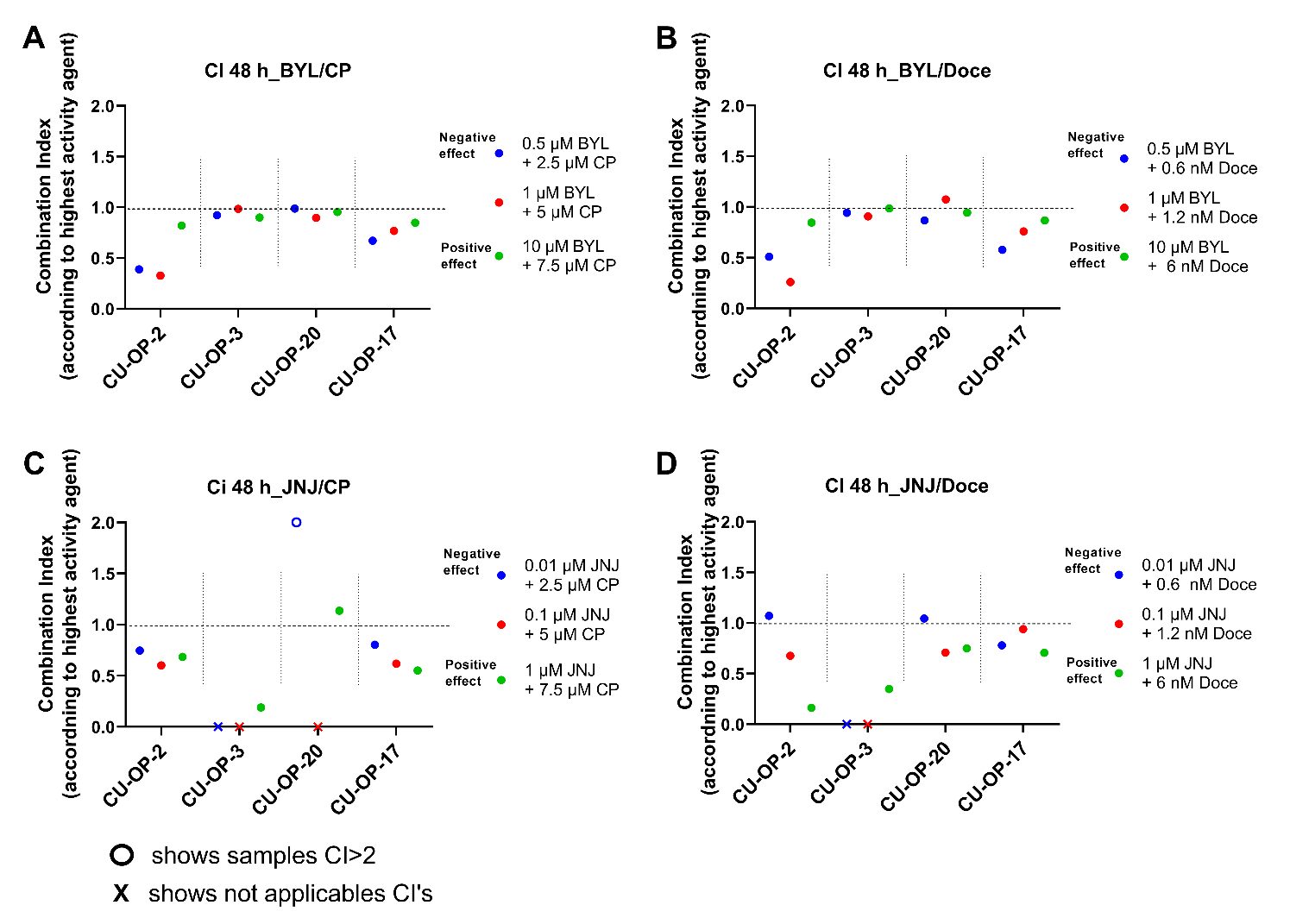


**Supplementary figure 1. Combinational effects of the PI3K inhibitor, BYL719, and FGFR inhibitor, JNJ-42756493, with cisplatin, and docetaxel on HPV^+^ CU-OP-2, -3, -20 and HPV^-^ CU-OP-17 cell lines after 48 h.** Combination indexes (CIs) were obtained by the highest single agent approach after treatment with BYL719 or JNJ-42756493 and cisplatin or docetaxel. Combination treatment of BYL with BYL719 or JNJ-42756493 resp. are shown in **(A)** and **(B)** resp., whereas combination treatment of JNJ-42756493 with the BYL719 or JNJ-42756493 resp. are shown in **(C)** and **(D)** resp. CIs were calculated from the mean of three experiments, analyzed by WST-1. CI denotes combination index; BYL denotes BYL719; JNJ denotes JNJ-42756493; CP denotes cisplatin; and DOCE denotes docetaxel. X denotes not applicable to show, so analysis could not proceed; o denotes CI>2, indicating a negative combination effect.


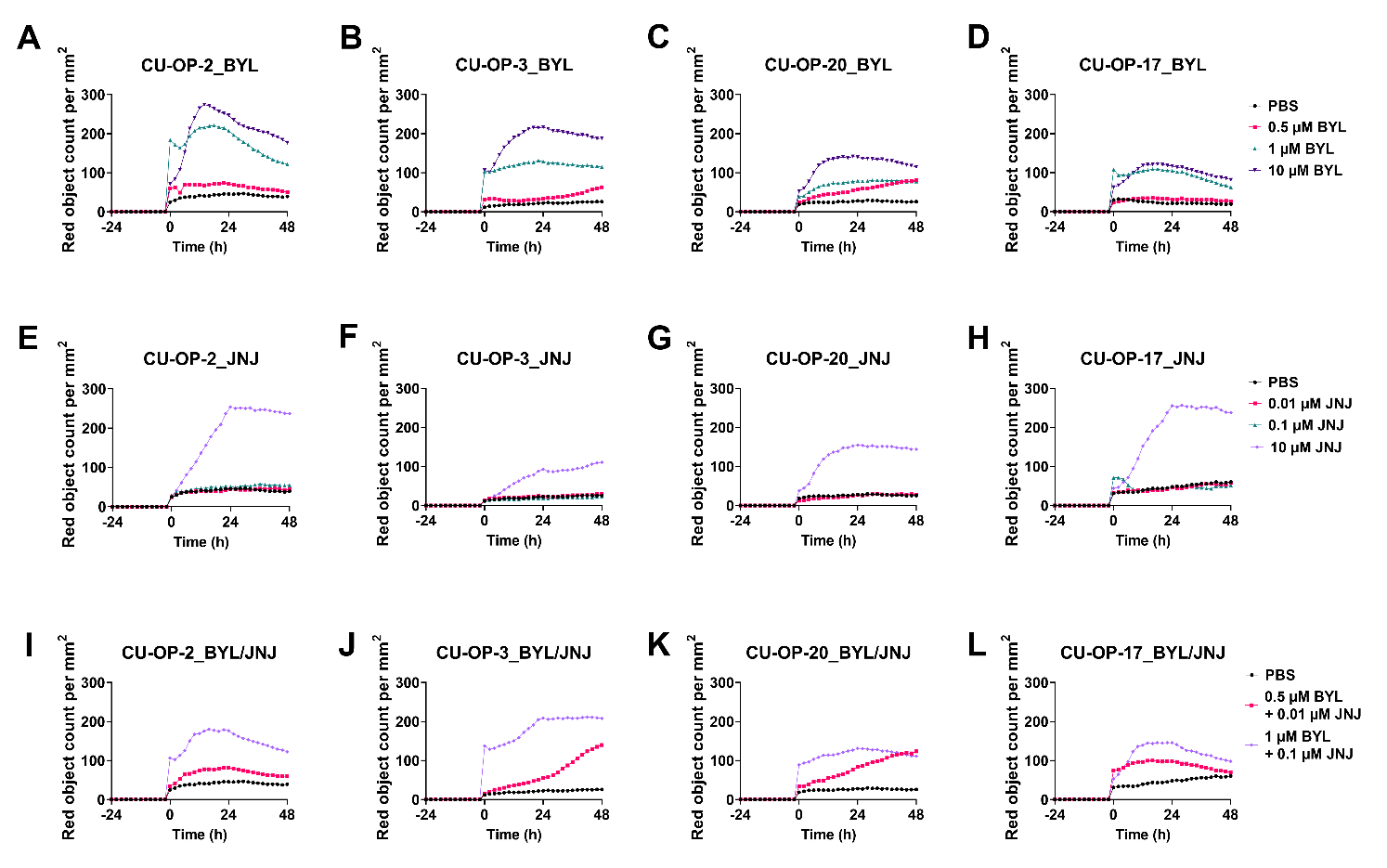


**Supplementary figure 2. Cytotoxicity response of HPV^+^ CU-OP-2, -3, -20 and HPV^-^ CU-OP-17 cell lines upon treatment with BYL719 and JNJ-42756493**. Cytotoxicity response of HPV^+^ CU-OP-2, -3, -20 and HPV^-^ CU-OP-17 to PI3K and FGFR inhibitors BYL719 **(A-D);** and JNJ-42756493 **(E-H);** and combinational treatment with BYL719 and JNJ-42756493 (I-L). The graphs represent three experimental runs per cell line. Red object count per mm2 denotes cytotoxicity response; BYL denotes BYL719; JNJ denotes JNJ-42756493; CP denotes cisplatin; and DOCE denotes docetaxel.


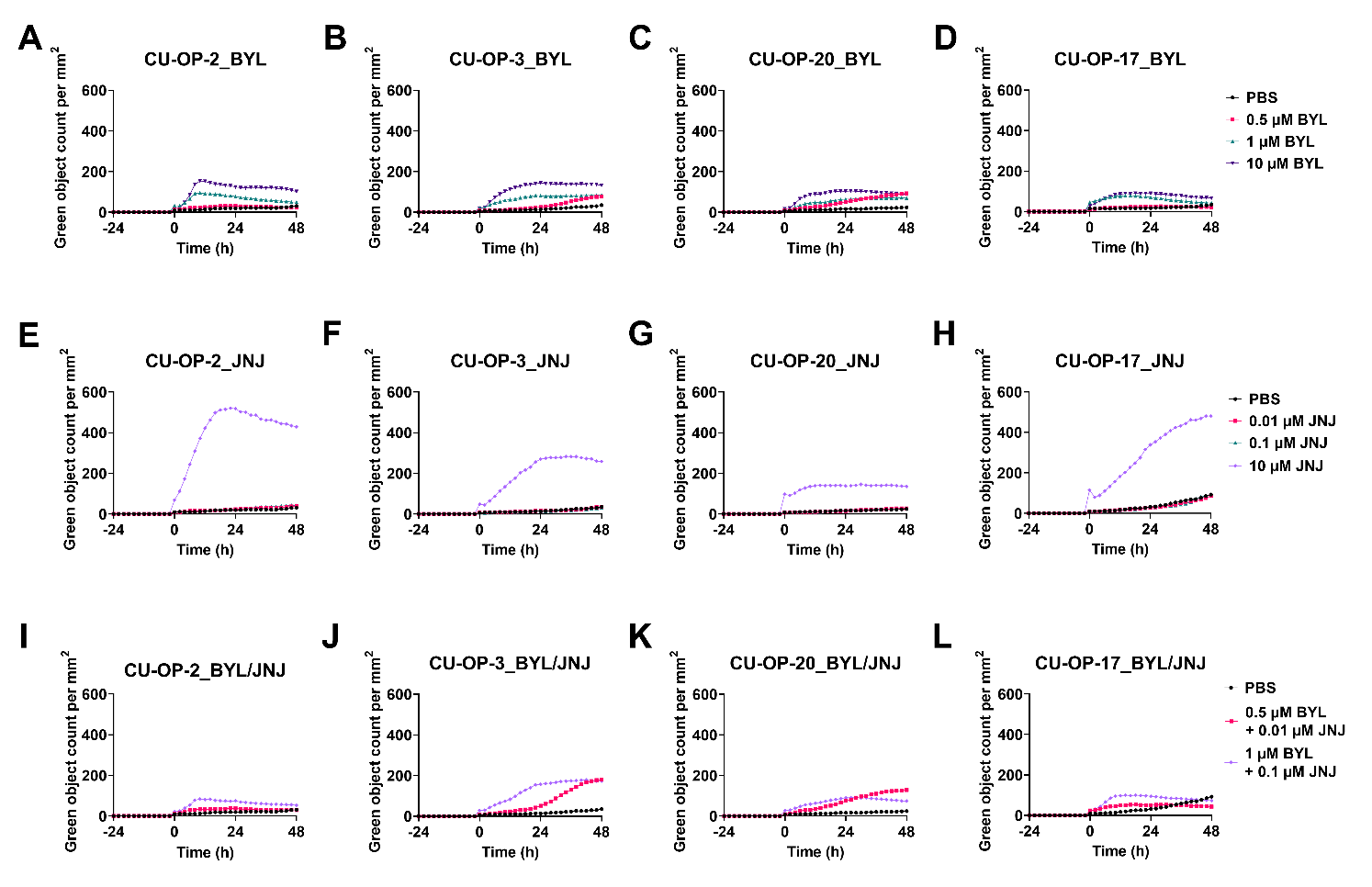


**Supplementary figure 3. Apoptosis response of HPV^+^ CU-OP-2, -3, -20 and HPV^-^ CU-OP-17 cell lines upon treatment with BYL719 and JNJ-42756493.** Apoptosis response of HPV^+^ CU-OP-2, -3, -20 and HPV^-^ CU-OP-17 to PI3K and FGFR inhibitors; BYL719 **(A-D)**; and JNJ-42756493 **(E-H)**; and combinational treatment with BYL719 and JNJ-42756493 (I-L). The graphs represent three experimental runs per cell line. Green object count per mm2 denotes apoptosis response; BYL denotes BYL719; JNJ denotes JNJ-42756493; CP denotes cisplatin; and DOCE denotes docetaxel.
